# Prevalence of *Chlamydia trachomatis* Genital Infection Among Persons Aged 14–39 Years — United States, 2007–2012

**Published:** 2014-09-26

**Authors:** Elizabeth Torrone, John Papp, Hillard Weinstock

**Affiliations:** 1 Division of STD Prevention, National Center for HIV/AIDS, Viral Hepatitis, STD, and TB Prevention, CDC

Infection with the bacterium*, Chlamydia trachomatis* (often termed “chlamydia”) is the most frequently reported sexually transmitted infection in the United States. The urethra is the most common site of infection in males, and the urethra and cervix are most commonly infected in females. Ascending infection in females can cause pelvic inflammatory disease, which can lead to infertility and ectopic pregnancy (1). Genital chlamydial infections are usually asymptomatic, and screening is necessary to identify most infections. Currently, chlamydia screening for sexually active women aged <25 years is recommended by the U.S. Preventive Services Task Force (grade B recommendation) (2). Chlamydia is nationally notifiable (3); however, if females do not access care or clinicians do not screen, many infections go undiagnosed, unreported, and untreated. CDC monitors population prevalence of genital chlamydial infection through the National Health and Nutrition Examination Survey (NHANES), which tests a sample of the U.S. population aged 14–39 years for genital *C. trachomatis* and found that the overall chlamydia burden in the United States decreased during 1999–2008 (7). Using data from the most recent cycles of NHANES (2007–2012), CDC estimated chlamydia prevalence among persons aged 14–39 years overall and by demographic characteristics and sexual behaviors. The prevalence of chlamydia among persons aged 14–39 years was 1.7% (95% confidence interval [CI] = 1.4%–2.0%). Chlamydia prevalence varied by age and race/ethnicity, with prevalence highest among non-Hispanic blacks (5.2%). Among sexually active females aged 14–24 years, the population targeted for routine screening, chlamydia prevalence was 4.7% overall and 13.5% among non-Hispanic black females. As chlamydia is common and infections are usually asymptomatic, health care providers should routinely screen sexually active young women aged <25 years for chlamydial infection, provide prompt treatment for infected persons, and ensure that infected patients’ sex partners receive timely treatment to prevent reinfection.

NHANES is a cross-sectional, complex, multistage survey designed to be nationally representative of the noninstitutionalized U.S. civilian population.* Participants are interviewed in person and are medically examined. During the examination, answers to sensitive questions on sexual and other behaviors are also collected using an audio and computer self-interview. During NHANES 2007–2012, a total of 8,827 persons aged 14–39 years were interviewed, and 8,563 were examined, for an overall response rate of 75%. Of those examined, 8,330 (97%) provided a urine sample that was tested for *C. trachomatis* using the Hologic/Gen-Probe Aptima nucleic acid amplification test. Nucleic acid testing of urine specimens detects urethral infection in males and both urethral and cervical infection in females. Prevalence of chlamydial infection and CIs were estimated by demographic and health care–related characteristics. Participants who responded “yes” to the question, “Have you ever had vaginal, anal, or oral sex” during the audio and computer self-interview were considered to be sexually active; chlamydia prevalence was estimated among this subset of respondents. Prevalence among sexually active females was estimated by age and race/ethnicity and by current use of oral contraceptives or a long-acting injectable contraceptive (i.e., DepoProvera). Prevalence ratios (PRs) and CIs were calculated to assess relative differences in prevalence. Difference in overall prevalence by NHANES cycle was assessed by the Rao-Scott chi-square test. All estimates were weighted to be nationally representative of the U.S. population, accounting for unequal probabilities of selection and nonresponse. Population counts were estimated by multiplying weighted prevalence estimates by the average of the Current Population Survey estimates during the three NHANES cycles (2007–2008, 2009–2010, and 2011–2012).

Among participants aged 14–39 years, overall chlamydia prevalence was 1.7% (CI = 1.4%–2.0%) suggesting that there are approximately 1.8 million prevalent infections nationally (CI = 1.4–2.1 million) (Table). Genital chlamydial infection was associated with age, race/ethnicity, income, marital status, number of sexual partners, and education. Prevalence of chlamydia among non-Hispanic blacks was approximately seven times the prevalence among non-Hispanic whites (PR = 6.7; CI = 4.3–10.6), and prevalence among Mexican-Americans was approximately three times the prevalence among non-Hispanic whites (PR = 2.9; CI = 1.7–5.1). Prevalence among sexually active participants who reported one sex partner in the last year was 1.4% (CI = 1.1%–1.7%), less than the 3.2% (CI = 2.2%–4.2%) prevalence among participants who reported two or more partners (PR = 0.4; CI = 0.3–0.7). Among sexually active female participants, prevalence was similar among women who were current users of oral contraceptives or DepoProva and women who were not using those birth control methods (PR = 0.8; CI = 0.4–1.6).

Among sexually active females, prevalence of chlamydia decreased with age (p<0.05) (Figure). Prevalence among sexually active females aged 14–24 years (the population targeted for chlamydia screening) was 4.7% overall (CI = 3.2%–6.1%) and varied by race/ethnicity (p<0.05) (Figure). Among sexually active females aged 14–24 years, approximately one in seven non-Hispanic black females was infected with chlamydia (prevalence = 13.5%; CI = 9.2%–17.7%); one in 22 Mexican-American females was infected (prevalence = 4.5%; CI = 2.6%–6.4%), and one in 55 non-Hispanic white females was infected (prevalence = 1.8%; CI = 0.3%–3.2%).

Overall prevalence of chlamydial infection among persons aged 14–39 years was similar over the three NHANES cycles combined for this analysis: 2007–2008: 1.6% (CI = 1.1%–2.2%); 2009–2010: 1.7% (CI = 1.2%–2.1%); and 2011–2012: 1.9% (CI = 1.5%–2.2%).

## Discussion

Chlamydia is the most commonly reported nationally notifiable disease, with over 1.4 million infections reported in 2012 (3). However, case reports likely underestimate the burden of disease because most infections are asymptomatic and are neither diagnosed nor reported. At the same time, because untreated chlamydia can persist, case report data are strongly influenced by screening activity, increasing with extensive screening and decreasing with limited screening. For these reasons, case report data are not reliable indicators of either population incidence or population prevalence. NHANES provides the best national estimate of chlamydia prevalence. The 2007–2012 NHANES indicate that an estimated 1.8 million persons aged 14–39 years in the United States have a genital chlamydial infection. Prevalence was highest among adolescents and young adults aged <25 years. Young persons might be at increased risk for infection because of biologic risk factors (e.g., cervical ectopy might predispose to infection and is more common in younger women), contextual risk factors (e.g., some young persons might lack power in relationships to insist upon condom use), or behavioral risk factors (e.g., younger persons might be more likely to have sex with new partners or sex with multiple partners) (1).

Although infection was more common among participants with multiple sex partners in the last year, prevalence among sexually active participants reporting only one partner in the last year was 1.4%, suggesting that not having had recent multiple partners does not eliminate risk for infection. Among sexually active females, use of oral contraceptives or DepoProvera was not associated with chlamydial infection, although use of these methods might be confounded by condom use because women using hormonal contraceptives might be less likely to use barrier contraceptives. Although previous studies have shown that use of hormonal contraceptives is associated with chlamydial infection (4), these studies were not population-based, and the hormonal contraceptives used were older formulations. Longitudinal studies using current formulations of contraceptives might be useful to better determine how contraceptive choice, including hormonal contraceptives and condom use, affects the acquisition of chlamydial infection.

Evidence suggests that chlamydia screening is cost-effective at prevalence >3% (5). Prevalence among sexually active young women aged 14–24 years was 4.7% overall, suggesting that routine screening of young women continues to be a cost-effective preventive intervention. However, in the United States, chlamydia screening rates are suboptimal, with fewer than half of sexually active young women screened annually. (6)

What is already known on this topic:

Chlamydia (*Chlamydia trachomatis* infection) is the most commonly reported notifiable disease in the United States, but case reports likely underestimate burden of disease because infections are usually asymptomatic and go undetected. Data from the 1999–2008 National Health and Nutrition Examination Surveys (NHANES) showed that chlamydia prevalence varies by age, sex, and race/ethnicity. Annual screening of sexually active women aged <25 years is recommended by the U.S. Preventive Services Task Force.

What is added by this report:

During NHANES 2007–2012, chlamydia prevalence was 1.7% among persons aged 14–39 years in the United States. Among sexually active females aged 14–24 years, chlamydia prevalence was 4.7% overall and 13.5% among non-Hispanic blacks.

What are the implications for public health practice:

High chlamydia prevalence among sexually active young females in the United States supports screening of all sexually active young females annually so that infected persons can be diagnosed and they and their sex partners can be treated promptly.

Similar to analyses of earlier NHANES data (7,8), this analysis found notable racial/ethnic disparities. Prevalence among sexually active, non-Hispanic black females aged 14–24 years was 13.5%, seven times the prevalence among white females (1.8%). Although the reasons for these racial/ethnic disparities are unknown, they might reflect different exposures to chlamydia because of differences in prevalence of chlamydia in sexual networks, as well as decreased access to routine preventive care that includes chlamydia screening and timely partner treatment. Effectively addressing disparities might require targeted interventions. In addition to requiring federally funded programs to focus efforts on populations with high burden of sexually transmitted infections, CDC currently funds the Community-Based Approaches to Reducing Sexually Transmitted Diseases (CARS) initiative to reduce disparities through implementation of interdisciplinary interventions, including facilitating enhanced community-clinical linkages to promote prevention and control of sexually transmitted infections.

The findings in this report are subject to at least four limitations. First, prevalence estimates do not include chlamydial infections at nongenital sites that can be infected through sexual contact, such as the rectum and oropharynx. Thus, prevalence estimates presented in this report are likely to underestimate the actual burden of sexually transmitted infection. Because rectal chlamydial infections might facilitate transmission of human immunodeficiency virus, further understanding of the prevalence of rectal infection is needed. Second, small sample sizes resulted in estimates with wide CIs. Although no temporal trend in prevalence was detected over the three survey cycles, the analysis likely was underpowered to detect epidemiologically significant changes in prevalence over time. Also because of small sample size, CDC was not able to provide estimates stratified by the sex of respondents or that of sex partners, or by both sex and age, except for estimates among sexually active young women, among whom prevalence is high. Third, some participants might have falsely reported being or not being sexually active. Finally, although the diagnostic tests for *C. trachomatis* used in NHANES are >95% sensitive and >99% specific, some results might be falsely positive or negative.

Currently, the U.S. Preventive Services Task Force recommends annual screening of all sexually active females aged <25 years and screening of older women at increased risk (e.g., women who have new or multiple sex partners) (2). Additionally, CDC recommends that men who report rectal sex should be screened at least annually and that targeted urogenital screening of sexually active young men in high-prevalence clinics might be considered (9). Treatment for chlamydia is simple and effective (9). However, reinfection is common, in part because of reinfection from an untreated partner (10). Clinicians should routinely screen young women and men who have sex with men for chlamydia and ensure that infected patients and their sex partners receive timely treatment to prevent reinfection (9). Strategies to increase screening in clinical facilities might include patient and clinician education and structural interventions at the health care facility, such as adding prompts to the electronic medical record (6). Timely treatment of sex partners might be facilitated by use of patient-delivered partner therapy, recommended by CDC for sexually transmitted chlamydial infection since 2006 (9).

## Figures and Tables

**FIGURE:**
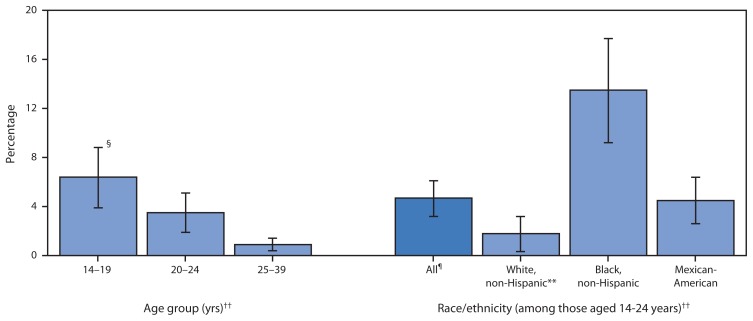
Prevalence of genital Chlamydia trachomatis* among sexually active^†^ females aged 14–39 years, by age group and race/ethnicity — National Health and Nutrition Examination Survey, United States, 2007–2012 * Prevalence estimates based urine specimen tested using the Hologic/Gen-Probe Aptima assay. ^†^ Among females who answered “yes” to the question, “Have you ever had vaginal, anal, or oral sex?” (n = 2,887). ^§^ 95% confidence interval. ^¶^ Data for persons of other racial/ethnic groups, including other, Hispanic (n = 492) and persons of multiple race/ethnicity (n = 422) are not presented but are included in overall estimate. ** Relative standard error >40% but <50% (n = 7). ^††^ Differences are statistically significant at p<0.05.

**TABLE:** Prevalence of genital Chlamydia trachomatis* infection among persons aged 14–39 years, by selected characteristics — National Health and Nutrition Examination Survey, United States, 2007–2012

Characteristic	Sample size	Prevalence (%)	(95% CI)	Prevalence ratio	(95% CI)
**Total**	**8,330**	**1.7**	**(1.4–2.0)**		
**Sex**					
Male	4,181	1.4	(1.1–1.8)	0.7	(0.5–1.1)
Female	4,149	2.0	(1.5–2.5)	1.0	
**Age group (yrs)**					
14–19	2,724	2.4	(1.7–3.1)	1.0	
20–24	1,456	2.9	(2.1–3.6)	1.2	(0.8–1.7)
25–39	4,150	1.1	(0.7–1.4)	0.4	(0.3–0.8)
**Race/Ethnicity** †					
Mexican-American	1,640	2.3	(1.4–3.1)	2.9	(1.7–5.1)
Black, non-Hispanic	1,887	5.2	(4.0–6.4)	6.7	(4.3–10.6)
White, non-Hispanic	3,019	0.8	(0.5–1.1)	1.0	
**Poverty-to-income ratio** §					
<100%	1,490	2.3	(1.5–3.0)	1.5	(1.1–2.0)
≥100%	3,615	1.6	(1.2–2.0)	1.0	
**Current health insurance** ¶					
Covered	5,753	1.6	(1.3–1.9)	0.8	(0.6–1.1)
Not covered	2,553	2.0	(1.5–2.5)	1.0	
**Education** **					
≤High school/GED	3,092	2.7	(2.1–3.4)	2.4	(1.6–3.6)
>High school/GED	3,371	1.1	(0.8–1.5)	1.0	
**Marital status** **					
Never married	2,131	2.3	(1.7–3.0)	2.8	(1.8–4.6)
Divorced/Widowed/Separated	429	3.0	(0.9–5.2)	3.7	(1.6–8.8)
Married/Living with Partner	3,043	0.8	(0.5–1.2)	1.0	
**Currently using oral contraceptives/DepoProvera** †† §§					
Yes	553	1.9	(0.7–3.1)	0.8	(0.4–1.6)
No	2,331	2.3	(1.7–3.0)	1.0	
**No. of sex partners in last year** §§					
0	402	1.8	(0.6–3.0)	0.6	(0.3–1.1)
1	3,727	1.4	(1.1–1.7)	0.4	(0.3–0.7)
≥2	1,686	3.2	(2.2–4.2)	1.0	
**Age at first sex** §§					
<14 yrs	779	2.6	(1.5–3.8)	1.4	(0.9–2.4)
≥14 yrs	5,062	1.8	(1.5–2.2)	1.0	
**Past STD diagnosis** §§ ¶¶					
Yes	579	1.9	(0.8–3.0)	0.8	(0.4–1.7)
No	1,564	2.3	(1.4–3.3)	1.0	

**Abbreviations:** CI = confidence interval; GED = General Education Development certification; STD = sexually transmitted disease.

* Prevalence estimates based urine specimen tested using the Hologic/Gen-Probe Aptima assay.

† Data for persons of other racial/ethnic groups, including other race, Hispanic (n = 925) and persons of multiple race/ethnicity (n = 859), are not presented but are included in overall analyses.

§ Ratio of family income to poverty level as defined by the U.S. Census Bureau.

¶ Based on response to the question, “Are you covered by health insurance or some other health care plan?”

** Among persons aged ≥18 years.

†† Among females.

§§ Among persons who answered “yes” to the question, “Have you ever had vaginal, anal, or oral sex?” (n = 5,848).

¶¶ Participants who have been told by a doctor or other health care professional in the last 12 months that they had chlamydia or gonorrhea or have ever been told they have herpes or genital warts.

## Footnotes

* Additional information available at http://www.cdc.gov/nchs/nhanes.htm.

## References

[1] Stamm WE, Holmes KK, Sparling PF, Stamm WE (2008). *Chlamydia trachomatis* infections in the adult. Sexually transmitted diseases.

[2] LeFevre ML (2014). Screening for chlamydia and gonorrhea: US Preventive Services Task Force recommendation statement. Ann Intern Med.

[3] CDC (2013). Sexually transmitted disease surveillance 2012.

[4] Mohllajee AP, Curtis KM, Martins SL, Peterson HB (2006). Hormonal contraceptive use and risk of sexually transmitted infections: a systematic review. Contraception.

[5] Marrazzo JM, Celum CL, Hillis SD, Fine D, DeLisle S, Handsfield HH (1997). Performance and cost-effectiveness of selective screening criteria for *Chlamydia trachomatis* infection in women. Implications for a national Chlamydia control strategy. Sex Transm Dis.

[6] Hoover KW, Leichliter JS, Torrone EA, Loosier PS, Gift TL, Tao G (2014). Chlamydia screening among females aged 15–21 years—multiple data sources, United States, 1999–2010. MMWR.

[7] Datta SD, Sternberg M, Johnson RE (2007). Gonorrhea and chlamydia in the United States among persons 14 to 39 years of age, 1999 to 2002. Ann Intern Med.

[8] Datta SD, Torrone E, Kruszon-Moran (2012). *Chlamydia trachomatis* trends in the United States among persons 14 to 39 years of age, 1999–2008. Sex Transm Dis.

[9] CDC (2010). Sexually transmitted diseases treatment guidelines, 2010. MMWR.

[10] Batteiger BE, Tu W, Ofner S (2010). Repeated *Chlamydia trachomatis* genital infections in adolescent women. J Infect Dis.

